# Assessment of epithelial barrier integrity in cellular monolayer models using low cost, clinically approved fluorescent contrast agents

**DOI:** 10.1371/journal.pone.0358746

**Published:** 2026-10-01

**Authors:** Elena Monfort Sanchez, Aaron F. Watson, Tafhima Haider, Despoina Chrysostomou, Maelle Storder, Nilanjan Mandal, Paul Kelly, Julian R. Marchesi, James Avery, Claire D. Bourke, Alex J. Thompson

**Affiliations:** 1 Helen Hamlyn Centre, Institute of Global Health Innovation, Department of Surgery & Cancer, Bessemer Building, Imperial College London, London, United Kingdom; 2 Blizard Institute, Queen Mary University of London, London, United Kingdom; 3 Tropical Gastroenterology and Nutrition Group, University of Zambia School of Medicine, Lusaka, Zambia; 4 Division of Digestive Diseases, Department of Metabolism, Digestion and Reproduction, St Mary’s Hospital, Imperial College London, London, United Kingdom; 5 School of Electronic and Electrical Engineering, University of Leeds, Woodhouse, Leeds, United Kingdom; 6 School of Infection & Immunity, College of Veterinary and Life Sciences, University of Glasgow, Sir Graeme Davies Building, 120 University Place, Glasgow, United Kingdom; Eötvös Loránd Research Network Biological Research Centre, HUNGARY

## Abstract

Epithelial barriers serve as the interface between body tissue and the external environment, maintaining tissue homeostasis by regulating the transport of various cells, molecules and microbes between these compartments. In particular, the epithelial barrier of the gut is a key component in numerous physiological processes that collectively define human health, such as nutrient absorption, microbiota regulation and neuroimmune communication. As such, gut epithelial barrier dysfunction, also known as ‘leaky gut’ syndrome has been associated with a variety of different conditions such as inflammatory bowel disease, type II diabetes, HIV and undernutrition. A diverse range of *in vitro* models have therefore been developed to better understand epithelial barrier function in different disease contexts. To study gut permeability *in vitro*, typical measurements include transepithelial electrical resistance (TEER) and the use of fluorescent contrast agents such as FITC-dextran to assess barrier integrity and permeability, with microfluidic ‘gut-on-a-chip’ models exclusively using FITC-dextran. However, utilising FITC-dextran in this context has multiple drawbacks for translational gastroenterology research. Namely, FITC-dextran is expensive, not clinically approved, and there is no agreed molecular weight ‘cut-off’ to determine barrier damage. The molecular weight of FITC-dextran also does not mimic substances used for barrier assessment in clinical settings (e.g., lactulose) nor the molecules thought to drive enteropathy *in vivo* (e.g., LPS and other pathogen associated molecular patterns (PAMPs)). Therefore, the clinically approved fluorescent contrast agents fluorescein and methylene blue (which have molecular weights comparable to substances used in the clinic) were explored in cellular monolayer models of the gut epithelium as low-cost alternatives for fluorescent assessment of epithelial barrier integrity. Both fluorescein and methylene blue exhibited analogous behavior to FITC-dextran, indicating suitability for use in fluorescent epithelial barrier assays. Hence, wider adoption of fluorescein and methylene may provide cost savings for *in vitro* epithelial barrier assessment assays as well as opportunities for more advanced, coherent and translational assessment of epithelial barrier function in health and disease.

## 1. Introduction

The gut epithelial barrier separates the internal contents of the intestine from the rest of the body and is involved in several crucial physiological processes that underpin human health, such as nutrient absorption, microbiota regulation, neuroimmune communication, as well as active and passive immunity [1–3]. Disruption of the epithelial barrier occurs in multiple human diseases and can range from sub-clinical increases in epithelial permeability due to reductions in tight junction proteins between epithelial cells to full breaches in the barrier due to physical damage or severe immunopathology. Intestinal epithelial barrier dysfunction is associated with leakage of blood and tissue into the gut lumen and increased transport of microbial and dietary antigens from the lumen into the lamina propria, which can enter circulation to drive systemic inflammation [4]. As such, a broad range of chronic inflammatory diseases including, liver inflammation, celiac disease, type II diabetes, HIV and neurodegenerative disorders such as Parkinson’s and Alzheimer’s disease, are associated with a so-called ‘leaky gut’ phenotype [3–6]. Thus, various models *in vitro* have been developed and deployed to further understand the underlying mechanisms that contribute to gut barrier impairment in a variety of disease contexts [7].

Cell culture monolayers utilising the human colorectal adenocarcinoma cell line Caco-2, using a cell culture insert-based system, have been extensively reported in the literature as a 2D *in vitro* model to study colonic barrier dysfunction. As a result, Caco-2 monolayers grown on cell culture inserts is widely considered the ‘gold standard’ for transport and barrier studies [8]. The utility of this model is largely made possible by the simplicity of introducing a transepithelial electrical resistance (TEER) electrode to measure tight junction integrity to validate stable monolayer formation [8]. Alternatively, Madin-Darby canine kidney (MDCK) cells have also been used to generate epithelial monolayers [9]. Although MDCK cells are derived from the canine kidney they offer a robust, reproducible and physiologically relevant model that is often used as a complementary model alongside human intestinal epithelial cell lines such as Caco-2 and T84. MDCK cells can serve this purpose as they form well developed tight junctions and are highly polarized when in culture, forming distinct apical and basolateral compartments imperative for transport and barrier studies. Since MDCK cells can produce stable monolayers within 3–4 days (rather than 14–21 days in their Caco-2 counterpart), MDCK cells have been adopted to improve throughput of barrier integrity studies within short culture periods [10]. While both Caco-2 and MDCK cell lines produce stable epithelial monolayers, these models lack the cellular heterogeneity typically found in the native gut microenvironment. To overcome this, co-culture and triple culture insert-based models (e.g., incorporating M cells and goblet cells to produce a mucus layer) have been utilised in various studies [11–14].

Significant advances have been made in developing more physiologically relevant *in vitro* models of the human gut epithelial barrier, using 2- and 3-dimensional cultures of primary stem cells from human biopsy and resection tissue. These ‘miniguts’ or organoids can generate the cellular diversity that epithelial cell lines lack, such as intestinal stem cells, enteroendocrine cells and Paneth cells [15]. These organoids also can retain their crypt-villus organization and more accurately mimic tight junction integrity and mucus uniformity [15]. Gut organoids can also be derived from patient-specific stem cells making them highly useful for gut barrier studies tailored for personalized medicine [15]. However, while gut organoids are established methods for improving the cellular heterogeneity of *in vitro* models, they have a number of limitations such as high cost and large heterogeneity between donors. Organoid models also remain difficult to culture for experiments that necessitate a high throughput. Nonetheless, the advantages of employing gut organoids to study gut barrier physiology have led to their assembly within cell culture inserts and in more advanced microfluidic ‘gut-on-a-chip’ models [15,16]. Organoid assembly in a culture insert advances on the above monolayer systems and is compatible with current TEER electrodes. However, because gut-on-a-chip models are typically complex and miniaturized, they are usually not compatible with TEER electrodes (although there have been attempts to integrate TEER electrodes within microfluidic chips [17]).

To address this issue and to allow an alternative assessment of epithelial barrier integrity, FITC-dextran is also commonly used to measure barrier permeability in both cell culture insert and microfluidic models [18,19]. FITC-dextran consists of the fluorophore fluorescein conjugated to dextran molecules of specific molecular weights. By introducing FITC-dextran to the apical (upper insert chamber) or endothelial channel (in gut-on-a-chip systems) sides of the epithelial barrier and measuring the fluorescence intensity of samples collected from the basolateral (well in a cell culture plate) or epithelial channel (gut-on-a-chip) sides, it is thus possible to further assess epithelial barrier integrity (i.e., by measuring apical-to-basal / endothelial-to-epithelial transmission of FITC-dextran across the epithelial barrier). This fluorescent methodology allows FITC-dextran to be used for assessment of a range of barrier defects (by using dextrans of varying molecular weights).

Despite FITC-dextran being able to be used for the assessment of a range of barrier dysfunctions, there are numerous drawbacks of using FITC-dextran for testing *in vitro*. Namely, FITC-dextran is expensive, not clinically approved and there is no consensus agreement to a molecular weight cut-off to determine barrier damage. Additionally, the molecular weights of FITC-dextrans are typically several kDa or higher, which does not correspond with the molecules used for barrier integrity assessment in humans. For example, the lactulose-mannitol (L:M) test is commonly deployed for gut barrier assessment *in vivo* and in humans [20,21]. Lactulose and mannitol are sugars with molecular weights of 342 g mol^-1^ and 182 g mol^-1^ respectively, much lower than the molecular weight of the smallest FITC-dextrans (minimum molecular weight ≈ 4000 g mol^-1^). These molecular weight discrepancies suggest that the permeation of FITC-dextran may not be a physiologically relevant assessment of gut barrier integrity.

Hence, to investigate development or more advanced and more physiologically relevant gut barrier integrity assays, this exploratory study involved a preliminary investigation of the permeation of three fluorescent contrast agents – fluorescein, FITC-dextran (4 kDa) and methylene blue – across MDCK cell monolayers. Contrast agent permeation was assessed in a insert-based system under various severities of epithelial barrier damage to investigate the potential use of fluorescent contrast agents with lower molecular weights than FITC-dextran for measurements of gut barrier integrity. An MDCK insert-based monolayer model was chosen for this purpose as it allowed formation of well-developed tight junctions over short culture periods, which was well suited to a pilot study investigating multiple contrast agents. Importantly, fluorescein and methylene blue have molecular weights comparable to lactulose and can therefore be expected to provide gut barrier damage readouts at physiologically relevant levels. Furthermore, both fluorescein and methylene blue are clinically approved and so can potentially be used to assess epithelial barrier integrity in both laboratory and clinical environments. Indeed, fluorescein was utilized for *in vivo* assessment of human gut barrier function in several recent studies [22–27]. Finally, both fluorescein and methylene blue are considerably cheaper than FITC-dextran offering the opportunity to reduce the costs of fluorescent epithelial barrier function assays, especially those involving ‘gut-on-a-chip’ approaches where use of FITC-dextran is necessitated [28].

## 2. Materals and methods

### 2.1. Cell culture

MDCK cells (provided by Professor Julian R. Marchesi, Imperial College London) were maintained in complete DMEM which constituted DMEM (Thermo) supplemented with 10% FBS (v/v, Thermo), 1% GlutaMAX (v/v, Thermo) and 1% penicillin-streptomycin (v/v, Thermo) and were passaged upon reaching 80% confluency. Cells were incubated in a 37°C, 5% CO_2_ humidified cell culture incubator.

### 2.2. Measurement of transepithelial electrical resistance in MDCK monolayers

When cultured on cell culture inserts, MDCK cells can polarize into apical and basolateral domains within a short time frame (3−4 days) allowing for the generation of 2D monolayers to test epithelial barrier integrity. MDCK cells were seeded onto cell culture inserts (pore size: 0.4 μm) in 24-well plates at a density of 4x10^5^ cells ml^−1^. Cells were maintained in complete DMEM medium and were used for experiments 4 days after seeding. Monolayer integrity was confirmed by TEER measurements taken with an EVOM3 Manual (World Precision Instruments). TEER was measured in Ohms cm^2^ and calculated as:


$$\begin{array}{c} {TEER}_{monolayer}=({TEER}_{sample}-{TEER}_{blank})\times A \end{array}$$ (1)

where TEER_blank_ is the raw TEER value measured in a blank cell culture well (with no cells but containing complete DMEM), TEER_sample_ is the raw TEER value measured in the sample (i.e., cell culture well with cells in complete cell culture medium), and *A* is the surface area of the cell culture insert (0.33 cm^2^).

Both apical and basolateral compartments of the 24-well cell culture insert system were filled with complete DMEM (200 µl in apical chamber, 1 ml in basolateral chamber). These DMEM volumes provided approximately equal liquid heights in the apical and basolateral compartments and also ensured that the medium in the basolateral compartment was in contact with the semi-permeable insert. This meant that the pressures of the two liquid columns were balanced and allowed for nutrient exchange between chambers.

### 2.3. Preparation of Ethanol, EGTA and fluorescent tracer solutions

Ethanol and EGTA (egtazic acid) were used to induce varying levels of epithelial barrier damage. Ethanol solutions at concentrations of 0.5%, 1%, 2%, 3%, 4%, 5% and 6% (v/v) were prepared by diluting absolute (≈ 100%) ethanol in DI water to 70% and then subsequently diluting 70% ethanol down to respective ethanol concentrations using complete DMEM. Cells were treated with the resulting ethanol solutions. EGTA was directly diluted in DMEM and used at concentrations of 0.25 mM, 0.5 mM, 1 mM, 2 mM, 4 mM, 8mM and 16 mM.

Fluorescein (Sigma), FITC-dextran (Sigma) and methylene blue (Sigma) solutions (concentrations shown in Table 1) were prepared by dissolving powdered dyes in complete DMEM medium prior to their addition onto MDCK monolayers. As the same concentration of different fluorescent tracers may yield different fluorescent intensities (e.g., due to differences in the fluorescence quantum yield), we calculated individual concentrations for each fluorescent contrast agent that would correspond to approximately the same fluorescence intensity across tracers. This was performed (prior to experiments) by standardising calculations to 100 μg mL^−1^ of FITC-dextran, an acceptable concentration in FITC-dextran flux assays. We first calculated the mass of FITC in 100 μg of FITC-dextran (based on the molar masses of FITC and glucose and the approximate FITC:glucose molar ratio in FITC-dextran reported by the supplier) as 0.835 μg. This was then chosen as the mass of fluorescein to give approximately equal fluorescence intensity to a mass of 100 μg of FITC-dextran.

**Table 1:** Fluorescent contrast agent concentrations applied to cell monolayers (i.e., concentrations in apical chambers) for each contrast agent (fluorescein, FITC-dextran and methylene blue) and each experimental concentration used (shown as percentages of maximal concentrations). All concentrations shown are in μg ml^-1^.

Contrast agent	Control	6.25%	25%	100%
FITC-dextran	0	6.25	25	100
Fluorescein	0	0.052	0.209	0.835
Methylene Blue	0	0.091	0.365	1.461

To calculate the equivalent mass for methylene blue, we then took the fluorescence quantum yield ($\Phi_{F}$) of fluorescein and methylene blue (as provided by the manufacturer: 0.91 for fluorescein, 0.52 for methylene blue) and calculated a correction factor (*C*) for methylene blue of $C=\Phi_{F}^{Methylene\;Blue}/\Phi_{F}^{Fluorescein}$. This accounted for the lower fluorescence quantum yield of methylene blue (relative to fluorescein) and yielded a mass of methylene blue of 1.461 μg. These masses then determined the maximum concentrations of FITC-dextran, fluorescein and methylene blue to be used in experiments (i.e., 100 μg ml^−1^ FITC-dextran, 0.835 μg ml^−1^ fluorescein, and 1.461 μg ml^-1^ methylene blue). These concentrations are hereafter referred to as 100% concentrations (as they were the maximal concentrations applied to MDCK monolayers). Two dilutions were also prepared for each contrast agent at equivalent concentrations of 6.25% and 25%. MDCK experiments were performed at all three contrast agent concentrations (6.25%, 25% and 100%) and under control conditions (with pure DMEM applied to the apical chamber containing no fluorescent contrast agent; referred to as 0% contrast agent concentration). The actual concentrations of each contrast agent (in the apical chamber) for each condition are presented in Table 1.

### 2.4. Permeability assay

To assess translocation of fluorescent contrast agents across the epithelial barrier, fluorescein (332.21 g mol^−1^), methylene blue (319.85 g mol^−1^) and FITC-dextran (4000 g mol^−1^) were added (individually) into the apical compartment of the insert-based system, either alone or in combination with ethanol or EGTA. Following addition of fluorescent contrast agents, the apical compartment contained 200 µl of fluorescent tracer mixed with complete DMEM, and the basolateral chamber contained 1 ml DMEM. As above, we observed that these volumes led to approximately equal liquid heights in the two chambers ensuring that the pressure of the liquid columns were balanced.

We note that a horizontal plate shaker (or other method to avoid an unstirred water layer) was not used in this study. As the chosen fluorescent contrast agents (fluorescein, FITC-dextran and methylene blue) are all highly soluble in aqueous media, an unstirred water layer and/or use of a horizontal plate shaker would not be expected to significantly affect results. Therefore, methodological steps to provide mechanical stirring were not used here.

Baseline samples (100 µl) were taken from the apical and basolateral chambers prior to addition of contrast agents and topped back up with 100 µl of complete DMEM. Following the addition of fluorescent agents, samples (100 µl) were collected from both chambers at 3 hours after dye addition (and the experiment was terminated after sampling). To enable fluorescence measurements, samples were transferred to new multiwell plates and fluorescence intensity was measured using a CLARIOstar fluorescence plate reader (BMG Labtech). Excitation and emission wavelengths used for fluorescence measurements for the three contrast agents are shown in Table 2.

**Table 2:** | Excitation and emission wavelengths used for fluorescence measurements of each fluorescent contrast agent (performed using CLARIOstar plate reader).

Contrast agent	Excitation (nm)	Emission (nm)
FITC-dextran	490	520
Fluorescein	490	520
Methylene Blue	665	686

TEER measurements were also recorded at baseline and at 1.5 and 3 hours. To account for variability across experiments, changes in TEER with respect to baseline (ΔTEER) were calculated in all cases as:


$$\begin{array}{c} \Delta TEER={TEER}_{monolayer}(t)-{TEER}_{monolayer}(t_{\text{0}}) \end{array}$$ (2)

where TEER_monolayer_(*t*) represents the absolute TEER value of the monolayer (calculated according to equation 1 above) at time *t* (i.e., at a measurement/sampling time point of 1.5 hours or 3 hours), and TEER_monolayer_(*t*_*0*_) represents the absolute TEER value of the monolayer at baseline (i.e., t = 0 hours).

Statistical analysis was performed in GraphPad Prism v8 using the Shapiro-Wilk test to assess normality followed by two-way ANOVA to compare the fluorescence intensities observed under different conditions (i.e., at different contrast agent and disruptor concentrations). The experimental procedure is illustrated in Fig 1A.

**Fig 1:**
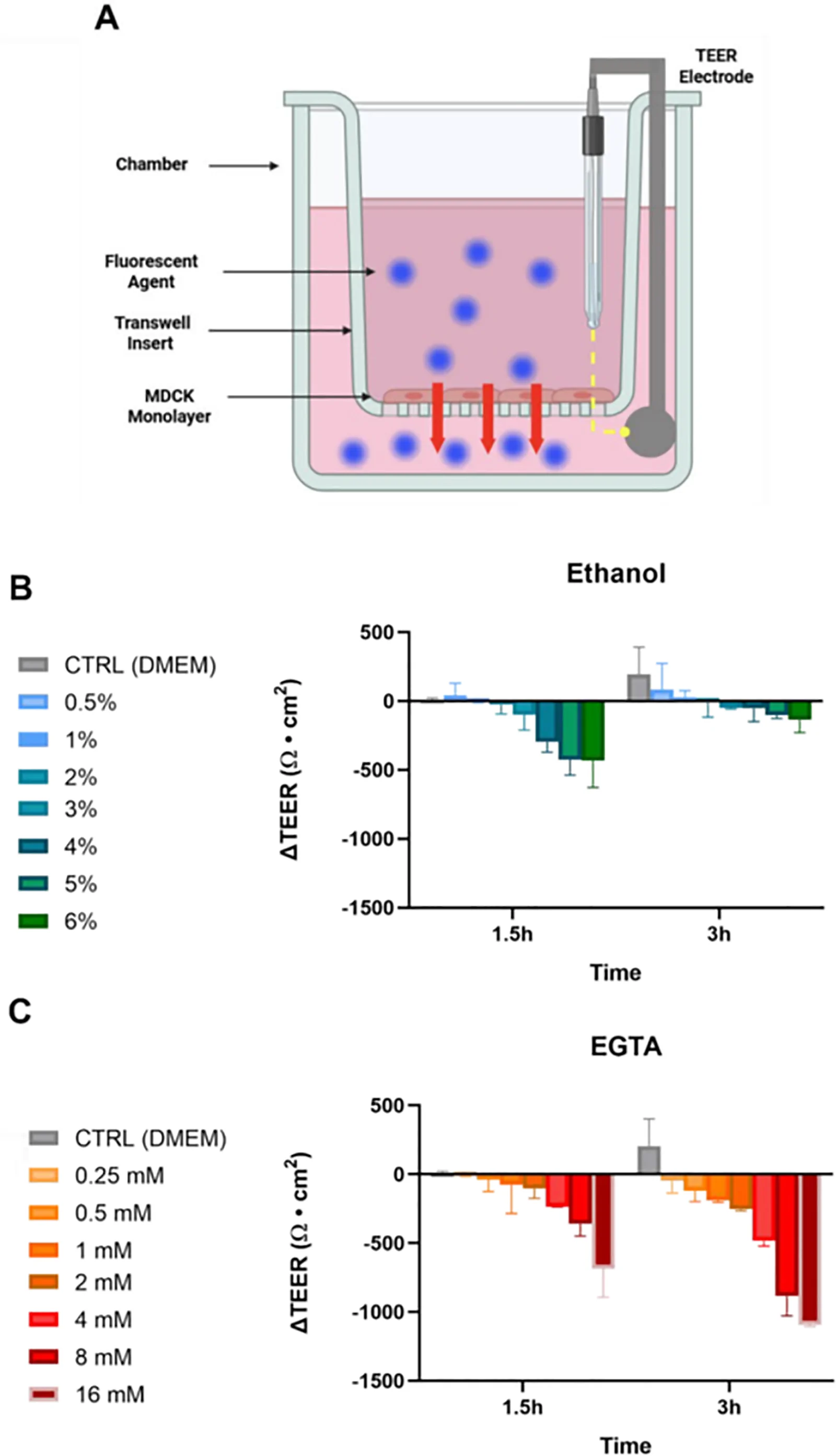
Optimization of epithelial barrier disruptor concentrations | (A) Schematic of the experimental apparatus used to measure MDCK monolayer integrity in a cell culture insert-based system. **(B-C)** Changes in TEER measured at 1.5 hours and 3 hours after the addition of different concentrations of **(B)** ethanol (v/v) and **(C)** EGTA. Two biological repeats (n = 2), one technical repeat (N = 1). Error bars shown on the figure represent the standard deviation.

## 3. Results

### 3.1. Confirmation of cell monolayer formation and optimization of epithelial barrier disruption

To study fluorescent contrast agent permeation across the epithelial barrier, a 2D cell culture insert system was deployed consisting of an MDCK monolayer cultured in the apical compartment of the insert-based system (Fig 1A). After 3 days, TEER measurements were taken to assess the establishment of MDCK monolayers prior to experimentation (Fig 1A). In this set of experiments monolayers that showed TEER readings greater than 1000 Ωcm^2^ were used. In this case TEER values of approximately 1500 Ωcm^2^ were observed in all wells, indicating the formation of monolayers with well-established tight junctions in all cases.

Ethanol and EGTA were applied to MDCK monolayers and TEER measurements were taken 1.5 hours and 3 hours post-exposure to optimise gut barrier disruption for downstream dye permeation experiments (Fig 1B,C). As expected, increasing the concentration of either ethanol or EGTA led to greater negative changes in TEER values (Fig 1B,C). Interestingly, the effect of ethanol after 3 hours is less pronounced than that observed after 1.5 hours (Fig 1B) and less pronounced than that observed with EGTA (Fig 1B,C). This difference was tentatively attributed to the evaporation of ethanol due to the small volumes used, leading to less barrier damage over time. The most dramatic change in gut barrier disruption was observed at 16 mM EGTA after 3 hours (Fig 1C).

### 3.2. Impact of Fluorescent contrast agents on epithelial barrier integrity

After validating which concentrations of barrier impairing agents effectively disrupt the MDCK monolayer, subsequent experiments explored the change in TEER value 3 hours after addition of fluorescein, FITC-dextran and methylene blue. These experiments were performed under control conditions (i.e., no barrier disruption) and in the presence of barrier disrupting agents (ethanol or EGTA) (Fig 2) to investigate any impact of the fluorescent contrast agents on epithelial barrier integrity. Concentrations of ethanol (v/v: 0.5%, 2% and 5%) and EGTA (0.5 mM, 2 mM and 8 mM) were chosen based on the experiments above (Fig 1 B,C) to provide a range of barrier damage severities.

**Fig 2:**
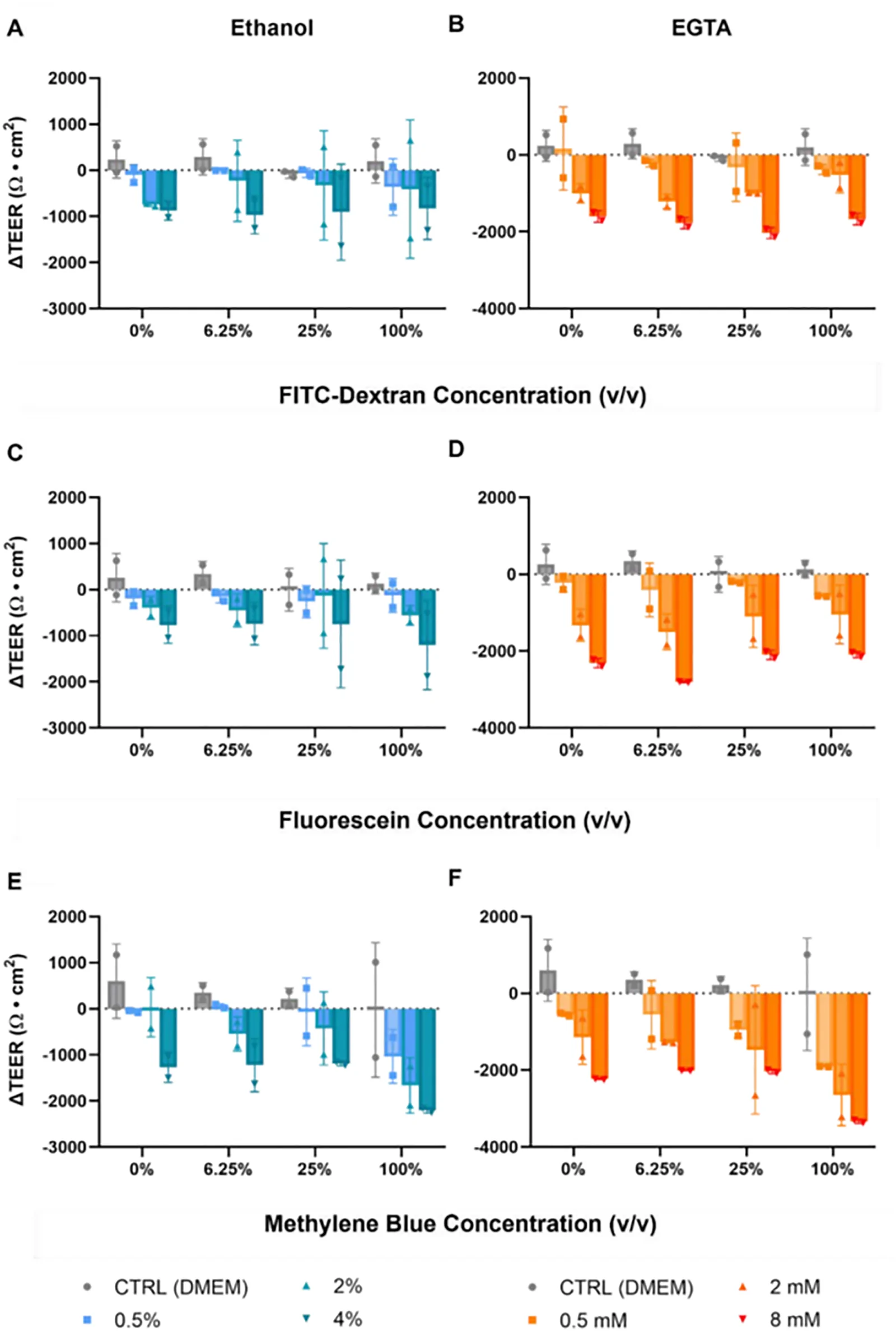
Assessment of epithelial barrier integrity using TEER for different concentrations of barrier disruptors (ethanol – left, EGTA – right) and for different fluorescent contrast agents. **(A-F)** Relative TEER changes (0-3 hours) for each fluorescent contrast agent with different levels of barrier disruption and different contrast agent concentrations. Control values reperesent TEER values for wells where no disruptor was applied. Two biological repeats (n = 2), one technical repeat (N = 1). Error bars represent standard deviations. Inset points shown alongside bars represent values from individual experiments.

As the severity of barrier damage increases (i.e., for increasing concentrations of ethanol and EGTA), a greater decrease in TEER value is observed for all fluorescent contrast agents and all contrast agent concentrations (Fig 2A**-**2F). This further demonstrates that higher concentrations of ethanol and EGTA induce greater degrees of epithelial barrier damage.

For fluorescein and FITC-dextran, the change in TEER value is approximately constant for all contrast agent concentrations (0–100%; see Fig 2A**-**2D). This data demonstrates that fluorescein and FITC-dextran (at the concentrations and incubations times used here) do not inherently damage the MDCK epithelial barrier (Fig 2 A-D). However, for methylene blue, TEER values appear to decrease for increasing methylene blue concentration (with no addition of ethanol/EGTA) (Fig 2E,2F). In addition a greater change in TEER value is observed at the highest methylene blue concentration (100%; 4.57 µM) for both barrier disrupting agents and for all disruptor concentrations (Fig 2,2F). This is likely to be the consequence of methylene blue toxicity on cell monolayers at higher concentrations [29]. Nonetheless, for all lower methylene blue concentrations tested here (25% and below; < 1.14 µM), changes in TEER values observed following addition of disruptors are approximately consistent across different dye concentrations and do not differ from those observed in control experiments (i.e., where no methylene blue was added; Fig 2E,2F).

Together, these results show that fluorescein and methylene blue (in addition to FITC-dextran) could be suitable fluorescent contrast agents for assessment of epithelial barrier integrity (albeit with methylene blue only being suitable at the lower concentrations used here) as their impact on epithelial barrier integrity is negligible compared to that of the barrier disrupting agents (ethanol and EGTA).

### 3.3. Fluorescein and methylene blue provide analogous readouts of epithelial barrier integrity to FITC-dextran

Permeation of fluorescent contrast agents across epithelial barriers was further explored to determine which dyes were able to effectively pass through MDCK monolayers under different severities of epithelial barrier damage. For all fluorescent contrast agents, the general trends observed are that fluorescence intensity increases with both increasing fluorescent dye concentration and increasing disruptor concentration (Fig 3).

**Fig 3:**
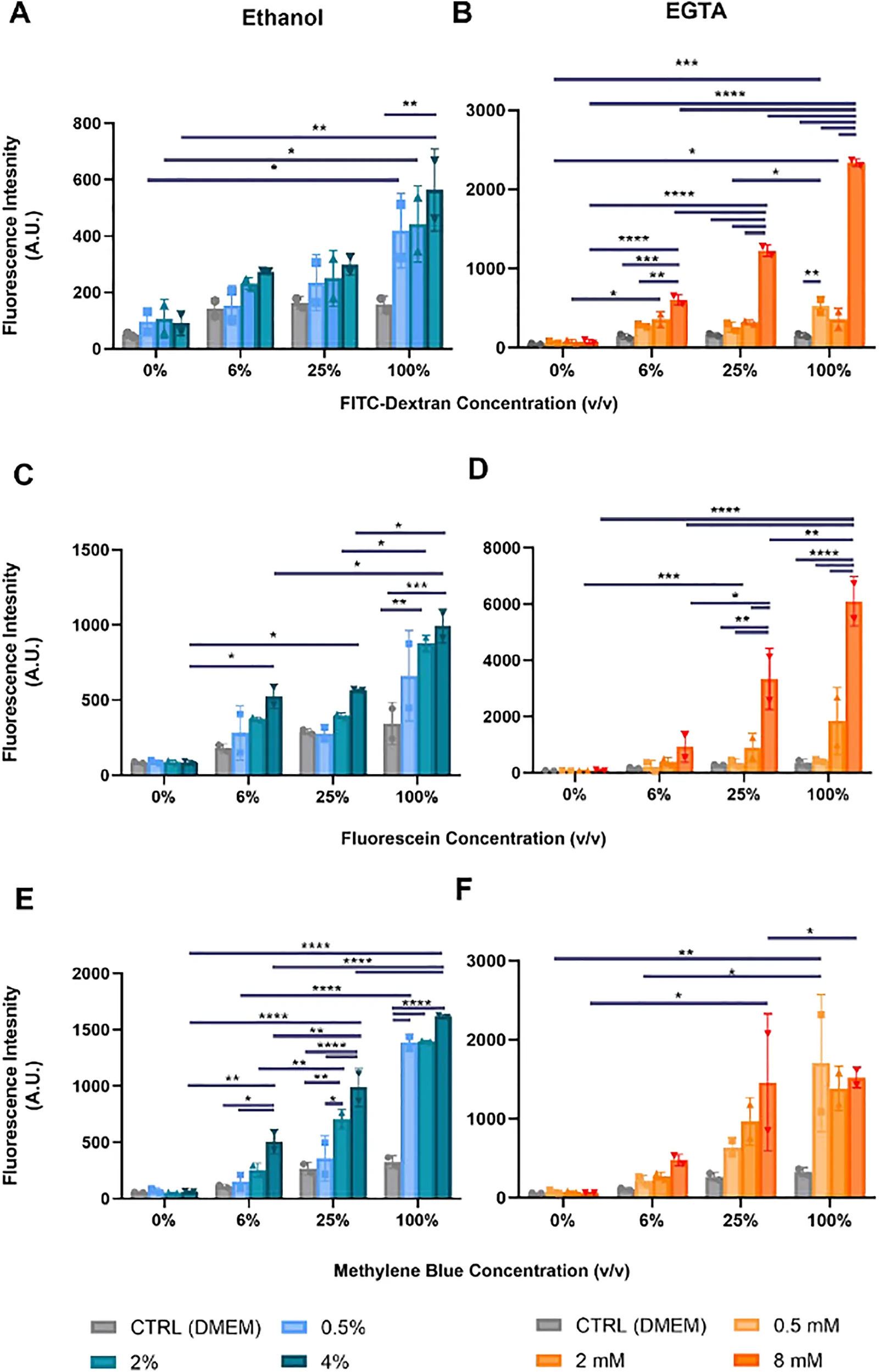
Permeation of fluorescent contrast agents across MDCK cell monolayers under different severities of epithelial barrier damage. **(A-F)** Fluorescence intensity recorded in basolateral compartments for each fluorescent contrast agent with different concentrations of ethanol **(left)** and EGTA **(right)** used to induce epithelial barrier damage. (**A, B**) fluorescein; (**C,D**) FITC-dextran; (**E, F**) methylene blue. Two biological repeats (n = 2), one technical repeat (N = 1). Data were statistically compared across different dye and disruptor concentrations using 2-way ANOVA. Asterisks indicate statistically significant differences with p < 0.05 (*), p < 0.01 (**), p < 0.001 (***) and p < 0.0001 (****). Error bars represent standard deviations. Inset points shown alongside bars represent values from individual experiments.

The Shaprio-Wilk test demonstrated normality for all but one condition (2 mM EGTA, FITC-Dextran). Therefore a two-way ANOVA was used for further statistical analysis. Importantly, fluorescence signals are lowest for the 0% contrast agent concentration in all cases (Fig 3), demonstrating that background signals (e.g., from fluorescent compounds in cell culture media) did not detrimentally affect our results. The most pronounced increases in fluorescence intensity are observed with 8 mM EGTA disruption (Fig 3B,D,F), in agreement with the results presented in Fig 1B,C (which show that 8 mM EGTA causes greater barrier disruption than any ethanol concentration at the 3 hour time point).

Interestingly, the trends observed for fluorescein (Fig 3A,B) are very similar to those observed for FITC-dextran (Fig 3C,D), but with higher fluorescence intensities measured using fluorescein (for all experimental conditions). This suggests that fluorescein can provide an *in vitro* readout of epithlial barrier damage in an analogous manner to FITC-dextran, but with a greater fraction of contrast agent crossing the epithelial barrier under all conditions. Interestingly, this may suggest that fluorescein could allow investigation of a wider range of barrier defects than FITC-dextran, by providing sensitivity to lower levels of barrier damage.

For methylene blue (Fig 3E,F), the trends observed are similar to those for both fluorescein and FITC-dextran (Fig 3A**-**3D) up to dye concentrations of 25%. At the highest methylene blue concentration (100%), the impact of ethanol and EGTA appears to be limited, with similar fluorescence intensities being measured at all disruptor concentrations (Fig 3E,F, 100% dye concentration). As discussed above, this was tentatively attributed to cellular toxicity of methylene blue at high concentrations as methylene blue has been shown to be toxic in a number of different cell lines [30,31^.^^32^]. In combination with ethanol or EGTA, the highest concentration of methylene blue appears to induce significant barrier disruption leading to observation of high fluorescence intensities for all disruptor concentrations. Despite this, at lower contrast agent concentrations (25% or below), methylene blue exhibited similar trends to those observed with FITC-dextran (compare Fig 3E,F against Fig 3C,D).

Hence, taken together, these results demonstrate that both fluorescein and methylene blue are promising candidates for monitoring of epithelial barrier integrity *in vitro*. Both dyes exhibit analogous permeation behaviour to FITC-dextran, which has been widely used for epithelial barrier assessment.

### 3.4. Permeation of all fluorescent contrast agents correlate with changes in TEER

Linear regression analysis was applied to the fluorescence intensities observed for fluorescein, FITC-dextran and methylene blue (at all contrast agent concentrations) to investigate the relationship between fluorescence intensity and change in TEER across different barrier disruption conditions (Fig 4; Supplementary Figures S1-S3 in S2 File). At a contrast agent concentration of 0%, correlation analysis demonstrates no change in fluorescence intensity despite changes in barrier integrity (as expected due to the lack of contrast agent; see Supplementary Figure S1 in S2 File). At the 6.25% contrast agent concentration, all fluorescent tracers show statistically significant correlations between fluorescence intensity and ΔTEER for both EGTA and ethanol disruption (Fig 4). Similar trends are observed for contrast agent concentrations of 25% and 100% (see Supplementary Figures S2 and S3 in S2 File). Together, this further validates methylene blue and fluorescein as potential markers of epithelial barrier integrity.

**Fig 4:**
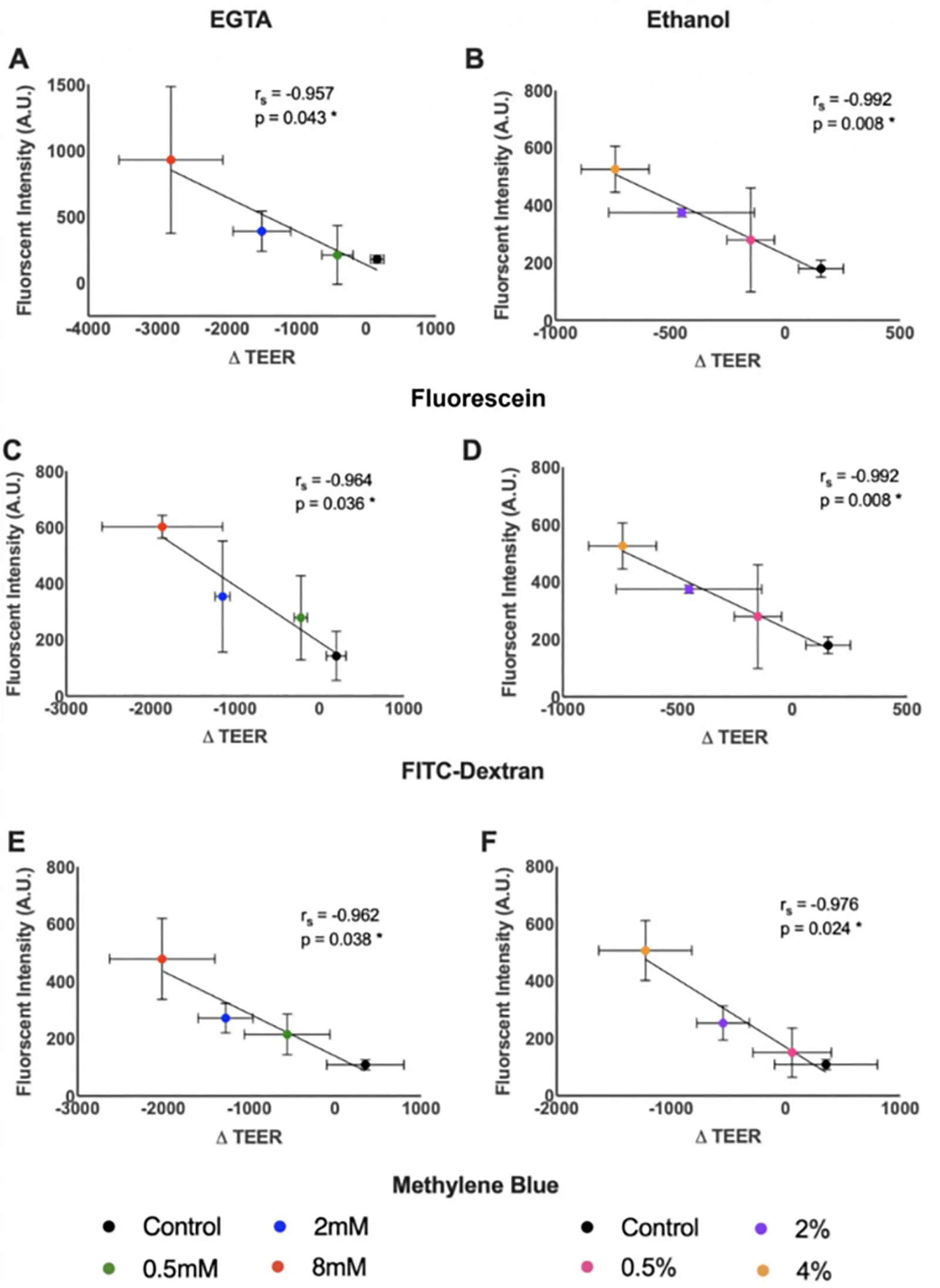
Regression analysis of fluorescence intensity and ΔTEER across various barrier disruptors at 6.25% dye concentration. Fluorescence intensity plotted against ΔTEER under EGTA (left) and ethanol (right) disruption. (A, B) Fluorescein; (C, D) FITC-dextran; (E, F) methylene blue. Two biological repeats (n = 2), one technical repeat (N = 1). Data was statistically analysed using Spearman’s Rank test. Correlation coefficients (r) and p-values are shown in their corresponding graphs. *p < 0.05. Error bars represent standard deviations.

## 4. Discussion and conclusions

In this exploratory study using *in vitro* epithelial barrier models, it is reported that fluorescein and methylene blue provide analogous readouts of epithelial barrier integrity to FITC-dextran, which is commonly used for gut epithelial barrier assessment in cellular, organoid and animal models. While results should be viewed as preliminary due to the low number of biological repeats, these findings have a number of important implications.

First, this data presents the opportunity to significantly reduce costs of current fluorescent permeability assays that use FITC-dextran (i.e., due to the lower cost and higher fluorescence quantum yields of fluorescein and methylene blue compared to FITC-dextran). Second, these findings indicate the possibility to explore physiologically relevant gut barrier defect sizes by assessing epithelial barrier integrity using fluorescent contrast agents *in vitro* that more closely mimic the sizes of the substances used *in vivo* for clinical assessment of gut barrier function (such as the sugars lactulose and mannitol used in the L:M test). Furthermore, as fluorescein and methylene blue are clinically approved contrast agents, these results also suggest the potential to assess epithelial barrier function in both laboratory and clinical settings using the same agents. This exciting advance would permit more coherent investigation of gut barrier function in health and disease by allowing comparable measurements to be made in cells, tissues, animals and humans. Indeed, this work also further validates the use of fluorescein for assessment of gut barrier function *in vivo*, as previously reported [21–27], as it demonstrates greater transmission of fluorescein across epithelial barriers under greater degrees of barrier disruption.

Third, and finally, these results indicate potential to explore multiple barrier defect sizes simultaneously by combining multiple fluorescent contrast agents with differing emission profiles and molecular weights. In particular, the results in this paper indicate that methylene blue and FITC-dextran could be used simultaneously (due to their differing fluorescence emission profiles) to provide a multifaceted assessment of epithelial barrier integrity.

The key limitations of this study include the use of an insert-based MDCK monolayer model (one of the simplest available epithelial barrier models), the investigation of a small number of fluorescent contrast agents, limits to the scope of the experiments performed (e.g., measuring contrast agent permeation at a maximum of two time points, studying response to two barrier disruptors, etc.), and a low number of biological replicates (n = 2). As such, future work will now entail extending this study to investigate behaviour of a larger number of contrast agents in more complex gut barrier models and under a wider range of barrier degradation conditions.

Importantly, the aim of this study was to further evaluate whether fluorescein and methylene blue can serve as fluorescent tracers of paracellular permeability analogously to FITC-dextran in *in vitro* barrier assays. As such, this preliminary study focused on measuring changes in TEER and fluorescence intensity of samples collected from basolateral chambers in addition to investigating TEER-fluorescence correlations. The data therefore served to demonstrate the potential of fluorescent contrast agent permeation as a proxy for epithelial barrier integrity.

Previous studies using fluorescence for *in vitro* epithelial barrier assessment have reported measurements of apparent permeability (P_app_), which provides a quantification of the mass of fluorescent contrast agent transported across the barrier [33–35]. Measurements of P_app_ enable comparison of data across studies but are still limited by variability in the integrity of the underlying monolayer (i.e., variability in baseline monolayer integrity will induce P_app_ variability across studies). In this study, we chose not to perform full P_app_ measurements (which require sample collection at multiple time points). Instead, simpler fluorescence intensity measurements at single time points were undertaken alongside TEER assessment to serve as proof-of-concept. While this does not enable direct comparison of fluorescent contrast agent transport against previous studies, it nonetheless allows preliminary demonstration of increased contrast agent transport across barriers under conditions of greater barrier damage.

This dual fluorescence-TEER approach has been used in previous studies, with significant inverse correlations having been reported between TEER and paracellular tracer flux in Caco-2 epithelial barrier models using probes including FITC-dextran and Lucifer Yellow [36–38]. Indeed, this dual approach has been applied *in vitro* under different contexts, for example to assess barrier changes induced by protein hydrolysates in the intestinal epithelium in Type 1 Diabetes [36]. Our pilot study followed a similar methodological approach to those investigations.

While FITC-dextran is one of the most widely used fluorescent tracers for cell monolayer permeability assessment, fluorescein has also previously been used for this purpose [39–41]. In this context, the comparable directional changes observed with all three fluorescent tracers (fluorescein, FITC-dextran and methylene blue) in relation to TEER serve to provide further validation of fluorescein as a permeability marker and proof-of-concept for methylene blue. To the best of our knowledge, methylene blue has not previously been reported as a validated fluorescent tracer for *in vitro* paracellular permeability assays in the wider literature. However, its behaviour has been directly compared with fluorescein in detecting tissue barrier leaks, where it performs similarly. For example, methylene blue and fluorescein have both been used to detect paracellular leaks in *ex vivo* porcine intestinal segments. Here, our preliminary data demonstrates similar behaviour *in vitro* between methylene blue and fluorescein further suggesting that methylene blue could be repurposed as a functional fluorescent contrast agent for *in vitro* studies investigating paracellular leakage.

A further limitation of the present study is that cytotoxicity was not directly assessed, and therefore the relative contributions of tight junction modulation versus epithelial cell loss to the observed decrease in TEER cannot be fully distinguished. However, epithelial barrier dysfunction *in vivo* is multifactorial and not solely driven by tight junction alterations. In conditions such as environmental enteropathy (EE) and inflammatory intestinal disorders, epithelial shedding and cell loss can also contribute to increased paracellular permeability [42,43]. In this context, the barrier disruption in the present model may reflect a combination of mechanisms including tight junction modulation and epithelial cell loss induced by ethanol or EGTA. While this may limit mechanistic resolution, it does not preclude functional assessment of paracellular permeability. Indeed, the primary aim of this study was to evaluate tracer flux under varying degrees of barrier disruption. The observed correlation between TEER reduction and increased tracer permeability supports the utility of this approach. Further studies incorporating direct measures of cell viability will nonetheless be important to further distinguish between the contributions of these mechanisms.

Importantly, however, the ethanol and EGTA concentrations used in this study were selected based on prior work demonstrating barrier modulation in epithelial monolayers without overt cytotoxicity. In particular, Ma et al. reported that exposure of filter-grown Caco-2 monolayers to ethanol concentrations of up to 10% (v/v) resulted in a dose-dependent decrease in TEER associated with disruption of the tight junction protein ZO-1, while lactate dehydrogenase (LDH) release assays indicated minimal cytotoxicity within this range [44]. On this basis, ethanol concentrations below 10% were selected in the present study to induce graded barrier disruption. Consistent with these findings, a concentration-dependent decrease in TEER was observed, with a slightly greater magnitude compared toobersvations made by Ma *et al*. (which may reflect differences between MDCK and Caco-2 models) [44].

Similarly, EGTA concentrations were chosen based on previous findings where Caco-2 monolayers were treated with EGTA concentrations ranging from 1–7 mM and exhibited up to a 60% decrease in TEER following treatment [43]. Interestingly, Panou et al. found that after 21 hours of recovery in cell culture medium TEER returned to baseline for EGTA concentrations ranging from 1–5 mM with ~90% recovery for Caco-2 monolayers treated with 7 mM EGTA [45]. This suggests that the lower EGTA concentrations used in this study (0.5 mM and 2 mM) are sufficient to disrupt tight junction integrity without inducing cytotoxicity, while the higher concentration (8 mM) is likely to cause only limited cytotoxicity. As such, all of the EGTA and ethanol concentrations used in this study are expected to induce tight junction disruption while causing only limited (or zero) cytotoxicity.

Overall, despite the limitations discussed above, this pilot study demonstrates that fluorescein and methylene blue – low-cost, clinically approved fluorescent contrast agents – can be used to assess epithelial barrier integrity in an analogous manner to FITC-dextran. This implies potential to dramatically reduce costs of *in vitro* experiments and to provide a range of exciting opportunities for more advanced and coherent investigation of epithelial barrier function in health and disease.

## Supporting information

S1 File Raw Data for Figures. Raw data made available for the figures within this manuscript.
(XLSX)

S2 File Supplementary Figures. Supplementary file containing supplementary figures S1-S3.
(DOCX)
