# Supplementary material for: Assessment of epithelial barrier integrity in cellular monolayer models using low cost, clinically approved fluorescent contrast agents
Source: PLoS One. 2026 Oct 1;21(10):e0358746. doi: 10.1371/journal.pone.0358746 (PMC13630259; doi:10.1371/journal.pone.0358746)
Supplement: Supplementary file 1 [file pone.0358746.s002.docx]

**SUPPLEMENTARY INFORMATION**

**Assessment of epithelial barrier integrity in cellular monolayer models using low cost, clinically approved fluorescent contrast agents**

Elena Monfort Sanchez^1†^, Aaron F. Watson^1†^, Tafhima Haider^2^, Despoina Chrysostomou^3^, Maelle Storder^1^, Nilanjan Mandal^1^, Paul Kelly^2,3^, Julian R. Marchesi^4^, James Avery^1,5^, Claire D. Bourke^2,6^, Alex J. Thompson^1*^

^1^Hamlyn Centre, Institute of Global Health Innovation, Department of Surgery & Cancer, Bessemer Building, Imperial College London, South Kensington, London, SW7 2AZ, UK

^2^Blizard Institute, Queen Mary University of London, London, E1 2AT, UK

^3^Tropical Gastroenterology and Nutrition Group, University of Zambia School of Medicine, Lusaka, Zambia

^4^Division of Digestive Diseases, Department of Metabolism, Digestion and Reproduction, St Mary's Hospital, Imperial College London, London, W2 1NY, UK

^5^School of Electronic and Electrical Engineering, University of Leeds, Woodhouse, Leeds, LS2 9JT, UK

^6^School of Infection & Immunity, College of Veterinary and Life Sciences, University of Glasgow, Sir Graeme Davies Building, 120 University Place, Glasgow G12 8TA, UK

^†^These authors contributed equally to this work.

^*^Corresponding Author: [alex.thompson08@imperial.ac.uk](mailto:alex.thompson08@imperial.ac.uk)

**This supplementary file includes:**

- Supplementary Figures S1-S3

**Figure S1.** **Regression analysis of fluorescence intensity and TEER across various barrier disruptors at 0% dye concentration**. Fluorescence intensity plotted against ΔTEER under EGTA (left) and ethanol (right) disruption. (A,B) Fluorescein; (C,D) FITC-dextran; (E,F) methylene blue. Two biological repeats (n=2), with one technical repeat (N=1). Data was statistically analyzed using Spearman’s Rank test. Correlation coefficients (r) and p-values are shown in their corresponding graphs. *p < 0.05. Error bars show standard deviation.

**Figure S2.** **Regression analysis of fluorescence intensity and TEER across various barrier disruptors at 25% dye concentration**. Fluorescence intensity plotted against ΔTEER under EGTA (left) and ethanol (right) disruption. (A,B) Fluorescein; (C,D) FITC-dextran; (E,F) methylene blue. Two biological repeats (n=2), with one technical repeat (N=1). Data was statistically analyzed using Spearman’s Rank test. Correlation coefficients (r) and p-values are shown in their corresponding graphs. *p < 0.05.  Error bars show standard deviation.

**Figure S3. Regression analysis of fluorescence intensity and TEER across various barrier disruptors at 100% dye concentration**. Fluorescence intensity plotted against ΔTEER under EGTA (left) and ethanol (right) disruption. (A, B) Fluorescein; (C, D) FITC-dextran; (E, F) methylene blue. Two biological repeats (n = 2), with one technical repeat (N=1). Data was statistically analyzed using Spearman’s Rank test. Correlation coefficients (r) and p-values are shown in their corresponding graphs. *p<0.05. Error bars show standard deviation.
