# Supplementary material for: Assessment of epithelial barrier integrity in cellular monolayer models using low cost, clinically approved fluorescent contrast agents
Source: PLoS One. 2026 Oct 1;21(10):e0358746. doi: 10.1371/journal.pone.0358746 (PMC13630259; doi:10.1371/journal.pone.0358746)
Supplement: Peer review history

# Peer review: Decision Letter 0

**DOI:** 10.1371/journal.pone.0358746.r001

**Authors:** Mária Deli

29 Nov 2025

Dear Dr. Watson,

Thank you for submitting your manuscript to PLOS ONE. After careful consideration, we feel that it has merit but does not fully meet PLOS ONE’s publication criteria as it currently stands. Therefore, we invite you to submit a revised version of the manuscript that addresses the points raised during the review process.

Please submit your revised manuscript by Jan 13 2026 11:59PM. If you will need more time than this to complete your revisions, please reply to this message or contact the journal office at plosone@plos.org. When you're ready to submit your revision, log on to https://www.editorialmanager.com/pone/ and select the 'Submissions Needing Revision' folder to locate your manuscript file.

- A rebuttal letter that responds to each point raised by the academic editor and reviewer(s). You should upload this letter as a separate file labeled 'Response to Reviewers'.
- A marked-up copy of your manuscript that highlights changes made to the original version. You should upload this as a separate file labeled 'Revised Manuscript with Track Changes'.
- An unmarked version of your revised paper without tracked changes. You should upload this as a separate file labeled 'Manuscript'.

If you would like to make changes to your financial disclosure, please include your updated statement in your cover letter. Guidelines for resubmitting your figure files are available below the reviewer comments at the end of this letter.

If applicable, we recommend that you deposit your laboratory protocols in protocols.io to enhance the reproducibility of your results. Protocols.io assigns your protocol its own identifier (DOI) so that it can be cited independently in the future. For instructions see: https://journals.plos.org/plosone/s/submission-guidelines#loc-laboratory-protocols. Additionally, PLOS ONE offers an option for publishing peer-reviewed Lab Protocol articles, which describe protocols hosted on protocols.io. Read more information on sharing protocols at https://plos.org/protocols?utm_medium=editorial-email&utm_source=authorletters&utm_campaign=protocols.

We look forward to receiving your revised manuscript.

Kind regards,

Mária A. Deli, M.D., Ph.D.

Academic Editor

PLOS ONE

Journal Requirements:

When submitting your revision, we need you to address these additional requirements.

1.Please ensure that your manuscript meets PLOS ONE's style requirements, including those for file naming. The PLOS ONE style templates can be found at

https://journals.plos.org/plosone/s/file?id=wjVg/PLOSOne_formatting_sample_main_body.pdf and

https://journals.plos.org/plosone/s/file?id=ba62/PLOSOne_formatting_sample_title_authors_affiliations.pdf

2. We note that the grant information you provided in the ‘Funding Information’ and ‘Financial Disclosure’ sections do not match.

When you resubmit, please ensure that you provide the correct grant numbers for the awards you received for your study in the ‘Funding Information’ section.

3. Thank you for stating the following financial disclosure:

“This article reports independent research supported by: the UK Medical Research Council

(MRC; grant number – MR/V012452/1); the National Institute for Health Research (NIHR) Imperial

Biomedical Research Centre (BRC); and the Imperial College London UKRI Impact Acceleration

Account supported by MRC (grant number – MR/X502959/1) and Rosetrees Trust. J.A.

acknowledges an Imperial College Research Fellowship. The views expressed in this publication

are those of the authors and not necessarily those of the NHS, the NIHR or the Department of

Health.”

Please state what role the funders took in the study.  If the funders had no role, please state: "The funders had no role in study design, data collection and analysis, decision to publish, or preparation of the manuscript."

If this statement is not correct you must amend it as needed.

Please include this amended Role of Funder statement in your cover letter; we will change the online submission form on your behalf.

4. Please note that funding information should not appear in any section or other areas of your manuscript. We will only publish funding information present in the Funding Statement section of the online submission form. Please remove any funding-related text from the manuscript.

5. Please include captions for your Supporting Information files at the end of your manuscript, and update any in-text citations to match accordingly. Please see our Supporting Information guidelines for more information: http://journals.plos.org/plosone/s/supporting-information

6. If the reviewer comments include a recommendation to cite specific previously published works, please review and evaluate these publications to determine whether they are relevant and should be cited. There is no requirement to cite these works unless the editor has indicated otherwise.

Additional Editor Comments:

Two experts gave detailed opinions on the manuscript. They agreed that the work has merits but both of them suggested amendments before acceptance. Please clarify and/or correct all the methodological points including repeat numbers (biological parallels or number of experiments?), TEER and Papp calculations

[Note: HTML markup is below. Please do not edit.]

Reviewer's Responses to Questions

**Comments to the Author**

1. Is the manuscript technically sound, and do the data support the conclusions?

Reviewer #1: No

Reviewer #2: Partly

**********

2. Has the statistical analysis been performed appropriately and rigorously?

Reviewer #1: No

Reviewer #2: No

**********

3. Have the authors made all data underlying the findings in their manuscript fully available?

The PLOS Data policy

Reviewer #1: Yes

Reviewer #2: Yes

**********

4. Is the manuscript presented in an intelligible fashion and written in standard English?

Reviewer #1: Yes

Reviewer #2: Yes

**********

Reviewer #1: This manuscript of Elena Monfort-Sanchez and Aaron F. Watson focuses on an important topic.

The gut barrier plays a crucial role in maintaining tissue homeostasis. Disruption of this barrier (commonly called “leaky gut”) is linked to several diseases, hence proper in vitro models and methods are crucial which are comparable with in vivo conditions. Authors conclude that fluorescein and methylene blue can effectively replace FITC-dextran in fluorescent assays of epithelial barrier function. However, the execution of the experiments, the interpretation of the results, and the conclusions drawn from them raise concerns and are not sufficiently convincing. Addressing these issues is crucial for the acceptance of the manuscript.

Minor comments:

1. Transwell is a brand name therefore it is written with capital first letter. If authors do not refer for the brand it is called cell culture insert or culture inserts. Please correct it where needed.

2. When presenting the results, please try to use identical scale divisions on the axes of graphs wherever possible, as this will make it easier to compare the results.

Major comments:

1. What is the reason for the difference in the number of biological repeats between Figures 2 and 3? Were the TEER measurements and the permeability assays not performed in the same experiment? My other concern is that 2 biological repeats and 1 technical repeat are not sufficient to draw reliable and robust conclusions. I would recommend repeating the experiments or including more biological replicates.

2. How can the fluorescence intensity data of this manuscript be compared with permeability data in the literature? It is suggested to calculate the Papp values (expressed as cm/s) to ensure comparability (e.g. Youdim et al, PMID: 14643163)

3. The discussion section is very short and does not compare the obtained results with literature data at all. I consider this as one of the weak points of the paper. Furthermore, fluorescein has been widely used by many research groups to assess the tightness of in vitro epithelial cell layers (e.g. Hellinger et al, PMID: 22906709)

Comments related to methods:

1. The authors should add the exact liquid volume used in the apical and basal compartment of the inserts. It is also important that the liquid levels in the two compartments are approximately at the same height to ensure that the pressure of the liquid columns is balanced.

2. It is not clear how ethanol treatment was performed. “Ethanol solutions at concentrations of 0.5%, 1%, 2%, 3%, 4%, 5% and 6% (v/v) were prepared by diluting absolute (≈ 100%) ethanol in DI water.” Why not in DMEM? Were the cells treated with DI water? Please clarify this.

3. Please describe how were the treatment concentrations of ethanol and EGTA determined? Were cytotoxicity tests (eg. MTT) performed beforehand to ensure that these concentrations do not cause cell death, but only the opening of the cell layer? Ethanol permeabilizes the plasma membrane of the cells, please add some sentences related to this effect in the Discussion.

4. “Fluorescein (Sigma), FITC-dextran (Sigma) and methylene blue (Sigma) stock solutions (concentrations shown in Table 1) were prepared by dissolving powdered dyes in sterilized DI water and diluting in complete DMEM medium prior to their addition onto MDCK monolayers.” Please describe the stock solution concentrations of the fluorescence dyes and the final concentration of DI water in cell treatment solutions.

5. “Contrast agent concentrations were chosen to give approximately equal fluorescence intensities for each agent at each concentration level as different fluorescent agents have different quantum yields.”

Was it calculated or measured prior the experiment? What are these concentrations based on? Please specify. Also please write the wavelengths to measure the fluorescent intensities. Please check the name of the instrument. Is it not Clariostar?

6. I have many concerns related to the permeability experiment.

6.1. It is recommended to use a horizontal plate shaker to avoid unstirred water layer (e.g. Youdim et al, PMID: 14643163)

6.2. Medium should be phenolred free, since it can interfere with fluorescence signal.

6.3. Please justify why 100 μL was taken out at 1.5h timepoint.

Every time you remove 100 µL from the donor, you change the donor concentration (and total donor volume). If donor concentration changes substantially, sink conditions are violated and you must correct for the changing donor concentration when computing permeability. Please clarify how was the permeability corrected for this.

Please calculate Papp values to reflect permeability. Without calculating Papp (which normalizes for concentration, area, and time), it’s difficult to directly compare results across experiments or with other studies.

7. The given raw TEER values are negative numbers. TEER values reflecting resistance can be zero but not negative numbers. If these data were calculated some other way, please provide the formula. Were cell-free inserts used for background TEER and also permeability measurements?

Comments related to results

Figure 1 and Figure 2. In my opinion, the interpretation of TEER results is not correct.

The Y-axis shows Ω×cm2, however, this is incorrect, as TEER values cannot be negative numbers. If the values were recalculated, please indicate the method used. I recommend presenting the values as a percentage of the control instead. If the values reflect relative changes this should be clearly described.

3.3 section

“The most pronounced increases in fluorescence intensity are observed with 8 mM EGTA disruption (Fig. 3B,D,F), in agreement with the results presented in Fig. 1B,C.” Only at figure 1C.

How was the fluorescent intensity calculated? How can these numbers be compared to the literature?

Reviewer #2: In the manuscript entitled “Assessment of epithelial barrier integrity in cellular monolayer models using low cost, clinically approved fluorescent contrast agents” the Authors describe the fluorescent method to measure the epithelial monolayer permeability using two markers: fluorescein and methylene blue. The idea of the study is reasonable and the results are promising. However, I have found some weak points that should be corrected before the manuscript is accepted for the publication in PLOS One.

Specific issues

Divide the “Methods” chapter into subsections.

Specify, what kind of TEER device was used with the necessary descriptions of the experimental conditions. How the TEER values were calculated?

What was the reason to use one-way ANOVA (parametric test) for the analysis? Was this decision supported by a normality test? If such a test was not performed, a non-parametric test (Kruskal-Wallis) should be used. The Authors have to perform the normality test to support the use of ANOVA or another parametric test for statistical analysis.

The bar charts are widely used in most scientific papers, but this is inappropriate in terms of statistics. Instead, a dot plot, boxes with whiskers, or the points showing the mean values with the error bars for standard deviation should be used. Please change the graph type in all the figures due to my suggestions.

The Authors have to specify if the error bars on the figures present the standard deviations (SD) or standard errors of the mean (SEM). From a substantive point of view the error bars should present the SD. If the error bars show the SEM, then it should be changed to SD.

Two biological repeats is not enough to perform a reliable statistical test. Three biological repeats is the absolute minimum. The Authors have to perform one more biological repeat wherever there are two biological repeats.

Editorial issues (page numbering applies to the entire Manuscript Draft document):

Correct the reference number 19 and 23. Number 19: “ACS Paper” – this information is not enough. Number 23: the reference is split into two paragraphs, thus merge these two paragraphs into one.

Page 11, line 5: change “However, While” to “However, while” (probably a typo).

English language comments

Page 10, third line in “Introduction”: to avoid unnecessary repetitions, change “and active and passive immunity” to “as well as active and passive immunity”.

The manuscript has to be resubmitted with the corrections according to the above suggestions, including the additional experiments to provide three biological repeats, and reconsidered again for publication.

**********

what does this mean?). If published, this will include your full peer review and any attached files.

If you choose “no”, your identity will remain anonymous but your review may still be made public.

**Do you want your identity to be public for this peer review?**  For information about this choice, including consent withdrawal, please see our Privacy Policy

Reviewer #1: No

Reviewer #2: No

**********

[NOTE: If reviewer comments were submitted as an attachment file, they will be attached to this email and accessible via the submission site. Please log into your account, locate the manuscript record, and check for the action link "View Attachments". If this link does not appear, there are no attachment files.]

To ensure your figures meet our technical requirements, please review our figure guidelines: https://journals.plos.org/plosone/s/figures

You may also use PLOS’s free figure tool, NAAS, to help you prepare publication quality figures: https://journals.plos.org/plosone/s/figures#loc-tools-for-figure-preparation.

NAAS will assess whether your figures meet our technical requirements by comparing each figure against our figure specifications.

# Author comment: Author response to Decision Letter 1

**DOI:** 10.1371/journal.pone.0358746.r002

1 Aug 2026

We thank the reviewers for taking the time to assess our manuscript and for their insightful comments. Please see below our responses to all of the reviewers’ comments. In this document, reviewer comments are shown in bold, black font and our responses are shown in green text. In our revised manuscript, all changes are highlighted using tracked changes.

Minor Comments:

1. Transwell is a brand name therefore it is written with capital first letter. If authors do not refer for the brand it is called cell culture insert or culture inserts. Please correct it where needed.

We thank the reviewer for highlighting this point. We have corrected all instances of the term ‘transwell’ to instead refer to ‘cell culture inserts.’

2. When presenting the results, please try to use identical scale divisions on the axes of graphs wherever possible, as this will make it easier to compare the results.

As suggested by the reviewer, we have adjusted y-axis scales to show equal scale divisions on all TEER graphs. For fluorescence graphs, we have chosen to retain different scales across graphs. This is because different fluorescent dyes and experimental conditions generate quite different fluorescence intensity values. Thus, plotting all graphs on identical scales makes some trends very hard to discern. For that reason, we have retained differing scales for fluorescence graphs to ensure that all experimental trends are easily observable and interpretable.

Major Comments (Results):

1. What is the reason for the difference in the number of biological repeats between Figures 2 and 3? Were the TEER measurements and the permeability assays not performed in the same experiment? My other concern is that 2 biological repeats and 1 technical repeat are not sufficient to draw reliable and robust conclusions. I would recommend repeating the experiments or including more biological replicates.

We thank the reviewing for highlighting this point. This was a typographical error within the original manuscript. TEER measurements and the permeability assays were indeed performed in the same experiments, and we performed 2 biological repeats and 1 technical repeat in all cases. This has been corrected in the updated manuscript.

We acknowledge that 2 biological repeats with 1 technical repeat are not ideal. However, due to a number of logistical considerations, we are unable to perform further experiments on this project (lead author has completed PhD, funding has expired, and author providing biological laboratory access has relocated). As such, we stress that the intention of our paper is to present a preliminary evaluation of the use of different yet potentially analogous fluorescent tracers to FITC-dextran for their suitability in gut transport assays. The intended interpretation of the data is to support preliminary methodological validation and tracer selection rather than making strong inferential/translational/mechanical claims. Although we only have two biological repeats, our results still capture meaningful, statistically significant trends that are appropriate for early-stage tracer selection and assay development. Hence, while preliminary, we believe the current data is suitable for publication and will help to enable future, larger scale studies to further guide choice of tracers and experimental parameters.

We have edited the text in numerous sections of the manuscript to acknowledge these points and to frame our results as “exploratory” and/or “proof-of-concept.”

2. How can the fluorescence intensity data of this manuscript be compared with permeability data in the literature? It is suggested to calculate the Papp values (expressed as cm/s) to ensure comparability (e.g. Youdim et al, PMID: 14643163)

While we agree that measurements of apparent permeability (Papp) would enable more direct comparison of our results against existing literature, we did not perform Papp measurements here. In addition, we are not able to calculate this retrospectively as we only recorded fluorescence data at a single time point in most experiments (accurate Papp measurements require multiple time points).

Despite this, we do not believe this detracts from the claims of our paper, which is focused on presenting proof-of-concept data supporting use of different/multiple fluorescent tracers for barrier function assessment. In that scenario, demonstration of higher fluorescence intensities at greater degrees of barrier disruption and inverse correlation between fluorescence intensity and TEER (which we have added to the updated manuscript) serve the purpose of our experiments As such, while calculation of Papp values would enhance cross-study comparability, it would not alter the results and trends observed in our pilot study showing TEER reduction and increased tracer flux at higher levels of barrier degradation.

Importantly, it is also noteworthy the Papp values are also dependent on the epithelial barrier itself (i.e. tighter barriers will naturally exhibit lower Papp values for any given fluorophore). This means that while Papp calculation would improve cross-study comparability, it would still have limitations in this regard (due to differences in baseline barrier conditions in different studies).

As such, we believe our results demonstrating changes in both TEER and fluorescence intensity with increasing barrier disruption (and inverse correlation between fluorescence intensity and TEER change) are appropriate for this investigation. These results clearly demonstrate that all fluorescent dyes investigated in this study pass MDCK epithelial barriers to greater degrees as barriers degrade. Thus, our exploratory data serves as proof-of-concept and supports further investigation of the use of multiple fluorescent contrast agents for assessment of epithelial barrier integrity.

We have added text into the Discussion & Conclusions section to address these points.

3. The discussion section is very short and does not compare the obtained results with literature data at all. I consider this as one of the weak points of the paper. Furthermore, fluorescein has been widely used by many research groups to assess the tightness of in vitro epithelial cell layers (e.g. Hellinger et al, PMID: 22906709)

As requested by the reviewer, we have incorporated additional text into the Discussion & Conclusions section to provide a more detailed comparison of our results against existing literature. As part of this, we acknowledged prior use of fluorescein and have cited the reference highlighted by the reviewer.

Major Comments (Methods):

1. The authors should add the exact liquid volume used in the apical and basal compartment of the inserts. It is also important that the liquid levels in the two compartments are approximately at the same height to ensure that the pressure of the liquid columns is balanced.

We have added the exact liquid volumes that were used in the apical and basolateral domains of the insert-based cell culture system. We have also noted that we observed these volumes led to equivalent heights of liquids in the apical and basolateral compartments (see Methods Sections 2.2 and 2.4).

2. It is not clear how ethanol treatment was performed. „Ethanol solutions at concentrations of 0.5%, 1%, 2%, 3%, 4%, 5% and 6% (v/v) were prepared by diluting absolute (≈ 100%) ethanol in DI water.” Why not in DMEM? Were the cells treated with DI water? Please clarify this.

We have clarified how the ethanol treatment was performed; we diluted absolute ethanol down to 70% using DI water and then diluted 70% ethanol down to the respective ethanol concentrations using complete DMEM. Cells were therefore effectively exposed to both ethanol and DI water in DMEM in all ethanol experiments, but with DMEM making up at least 90% (v/v) of the solutions in all cases. This approach was used to minimise evaporation of ethanol during the solution preparation process (by using a working ethanol concentration of 70% rather than 100%). We have clarified this process in the Methods section (see section 2.3).

3. Please describe how were the treatment concentrations of ethanol and EGTA determined? Were cytotoxicity tests (eg. MTT) performed beforehand to ensure that these concentrations do not cause cell death, but only the opening of the cell layer? Ethanol permeabilizes the plasma membrane of the cells, please add some sentences related to this effect in the Discussion.

We did not perform cytotoxicity tests such as the MTT assay as part of this pilot study. Instead, we chose EGTA and ethanol concentrations based on previous published work that would be expected to provide disruption of tight junctions without causing significant cytotoxicity. We have added additional text into the Discussion section to outline how the specified ethanol and EGTA concentrations were chosen. We have also specifically acknowledged the lack of cytotoxicity assays as a limitation of our study.

4. “Fluorescein (Sigma), FITC-dextran (Sigma) and methylene blue (Sigma) stock solutions (concentrations shown in Table 1) were prepared by dissolving powdered dyes in sterilized DI water and diluting in complete DMEM medium prior to their addition onto MDCK monolayers.” Please describe the stock solution concentrations of the fluorescence dyes and the final concentration of DI water in cell treatment solutions.

We have clarified that the fluorescent agents were not dissolved in DI water but rather were directly dissolved in complete DMEM. This was a mistake in our original manuscript and we thank the reviewer for highlighting this. As such, no DI water was applied to the cells as part of the fluorescent dye solutions. The concentration of the stock solutions and all dilutions are shown in Table 1 (see Methods section, section 2.3). The concentrations shown in Table 1 are the final concentrations applied to cells (i.e. the concentrations in the apical chamber). We have added further text into section 2.3 to clarify these points.

5. “Contrast agent concentrations were chosen to give approximately equal fluorescence intensities for each agent at each concentration level as different fluorescent agents have different quantum yields.” Was it calculated or measured prior the experiment? What are these concentrations based on? Please specify. Also please write the wavelengths to measure the fluorescent intensities. Please check the name of the instrument. Is it not Clariostar?

We have provided further detail to clarify our calculations of fluorescent contrast agent concentrations in the methods section (see section 2.3). As part of this, we have noted that these values were calculated prior to experiments (and then used in all subsequent experiments).

The name of the instrument has been corrected to ‘CLARIOstar’ (see section 2.4) – we thank the reviewer for highlighting this error. We have also listed the excitation and emission wavelengths used for each fluorescent contrast agent (see section 2.4 and Table 2), as requested by the reviewer.

6.1. It is recommended to use a horizontal plate shaker to avoid unstirred water layer (e.g. Youdim et al, PMID: 14643163)

A horizontal plate shaker was not used in the experiments presented here. As discussed above, we are not currently able to perform further experiments to incorporate this. Importantly, however, the unstirred water layer primarily affects highly lipophilic and rapidly permeating compounds. The fluorescent compounds used in this study are all highly soluble in aqueous media. As such, the unstirred water layer would be expected to have only a minimal effect on the permeation of the fluorescent agents used here (and so incorporating a horizontal plate shaker would not significantly alter our findings). Furthermore, as use of a horizontal plate shaker would act to speed up diffusion of fluorescent contrast agent in the apical chamber, any changes induced would only be expected to cause greater permeation of fluorescent tracers across the epithelial barrier (by accelerating transport towards the barrier). Thus, our data provides a sound proof-of-concept for translocation of all fluorescent agents across epithelial barriers and, if anything, our results may underestimate the degree of translocation in each case.

We have included additional text in the Methods section (section 2.4) to explain these points.

6.2. Medium should be phenol red free, since it can interfere with fluorescence signal.

We agree with the reviewer that use of phenol red free medium would have been optimal for this study. However, only standard DMEM (containing phenol red) was available to us in this pilot study. Importantly, phenol red interference is most problematic for low-intensity signals. Fluorescein and FITC-dextran exhibit very strong fluorescence at 490/520 nm excitation/emission, and methylene blue also has a strong absorbance that is distinct from phenol red background. Our results also show that fluorescence signals observed from our chosen fluorescent contrast agents are significantly higher than any background observed in the absence of exogenous fluorescent dyes – i.e. fluorescence signals from fluorescein, FITC-dextran and methylene blue are all high compared to any phenol red background (compare fluorescence intensities for 6.25%, 25% and 100% fluorescent dye concentrations against those for 0% concentration in Figure 3). This demonstrates that phenol red background signals did not detrimentally affect our results. We have added a sentence into the Results section (section 3.3) to make note of these points.

6.3. Please justify why 100 μL was taken out at 1.5h timepoint.

Every time you remove 100 µL from the donor, you change the donor concentration (and total donor volume). If donor concentration changes substantially, sink conditions are violated and you must correct for the changing donor concentration when computing permeability. Please clarify how was the permeability corrected for this. Please calculate Papp values to reflect permeability. Without calculating Papp (which normalizes for concentration, area, and time), it’s difficult to directly compare results across experiments or with other studies.

Samples for fluorescence measurement were only collected at 3 hours post-addition of fluorescent contrast agents. Baseline samples were collected prior to addition of contrast agent from both the apical and basolateral chambers, with the media topped back up by the same volume immediately after sample collection. Following the addition of fluorescent agents, 100 µl samples were collected from both chambers at a single time point (3 hours) after dye addition. Experiments were then terminated after sampling. As a result, it was not necessary to perform any correction to account for removal of fluorescent agent from the cell culture system. We have edited text in the Methods section to clarify our experimental protocol (see section 2.4) where we have also noted that TEER measurements were made at three time points (baseline, 1.5 hours and 3 hours). We thank the reviewer for highlighting this point, which was somewhat ambiguous in our original manuscript.

We also note again that the goal of our study was to show preliminary data demonstrating that fluorescein and methylene blue can be used to assess barrier function in an analogous manner to FITC-dextran. While we agree that measurements of apparent permeability can be useful in allowing comparison across studies, showing an inverse relationship between TEER and fluorescence is a widely accepted proxy in the literature and is appropriate to demonstrate proof-of-concept for fluorescent detection of barrier degradation. As discussed above, we have added text into the Discussion & Conclusions section to explain these points.

7. The given raw TEER values are negative numbers. TEER values reflecting resistance can be zero but not negative numbers. If these data were calculated some other way, please provide the formula. Were cell-free inserts used for background TEER and also permeability measurements?

figure 1 and Figure 2. In my opinion, the interpretation of TEER results is not correct. The Y-axis shows Ω×cm2, however, this is incorrect, as TEER values cannot be negative numbers. If the values were recalculated, please indicate the method used. I recommend presenting the values as a percentage of the control instead. If the values reflect relative changes this should be clearly described.

We thank the reviewer for highlighting this inconsistency. All TEER values presented in Figures 1 and 2 represent changes in TEER relative to baseline measurements (i.e. ΔTEER) and not absolute TEER values. As such, values are generally negative as we observed decreases in TEER following addition of disruptors EGTA and ethanol (although we note that some positive values are observed under control conditions where monolayers continued to grow between baseline and measurement/sampling time points). We have now added text into the Methods section to clearly describe how TEER values were calculated (both absolute values and ΔTEER values) – see sections 2.2 and 2.4, and equations (1) and (2).

3.3section

“The most pronounced increases in fluorescence intensity are observed with 8 mM EGTA disruption (Fig. 3B,D,F), in agreement with the results presented in Fig. 1B,C.” Only at figure1C. How was the fluorescent intensity calculated? How can these numbers be compared to the literature?

In this section (3.3), we have added text to the paragraph highlighted by the reviewer to more clearly explain the reference to Figure 1B,C (where we in fact compare against results for both ethanol (Fig. 1B) and EGTA disruption (Fig. 1C).

To measure fluorescence intensity, a 96-well plate containing the 100 µl samples was placed in a CLARIOstar fluorescence plate reader at the set excitation and emission wavelengths of the tracer (see Table 2, section 2.4) and the resulting fluorescence intensity values were plotted. Importantly, as discussed above, these numbers are not to be cross-compared with literature but rather to show internal consistency that higher fluorescence intensity is observed at greater levels of barrier disruption and that higher fluorescence intensity correlates with greater decreases in TEER.

We have amended the Methods text in section 2.4 to more clearly explain how fluorescence measurements were performed.

Divide the “Methods” chapter into subsections.

We have divided the Methods section into subsections as requested by the reviewer.

Specify, what kind of TEER device was used with the necessary descriptions of the experimental conditions. How were the TEER values calculated?

The TEER device used was the EVOM3 Manual by World Precision Instruments. To measure TEER values, we first measured absolute TEER values on both the samples (i.e. cell culture inserts with MDCK monolayers) and blank inserts containing cell culture medium only. The monolayer TEER value was then calculated by subtracting the blank value from the sample value (see equation (1), section 2.2). To assess changes in response to ethanol or EGTA disruption, we then calculated changes in TEER relative to baseline (see equation (2), section 2.4). The resulting ΔTEER values are presented in Figures 1 and 2.

We have included additional text in sections 2.2 and 2.4 of the Methods to further explain these points.

What was the reason to use one-way ANOVA (parametric test) for the analysis? Was this decision supported by a normality test? If such a test was not performed, a non-parametric test (Kruskal-Wallis) should be used. The Authors have to perform the normality test to support the use of ANOVA or another parametric test for statistical analysis.

We thank the reviewer for highlighting this point and we clarify that a two-way ANOVA was used for the analysis. The Shapiro-Wilk test was used to assess normality and all conditions passed normality but one (2 mM EGTA, FITC-Dextran). Given this result, and given the preliminary nature of our data (i.e. two measurements per condition), we chose to use two-way ANOVA for all further statistical analysis. While the size of our dataset means that all results should be considered exploratory at this stage (regardless of significance level), use of two-way ANOVA was nonetheless informative in guiding interpretation of results and was appropriate to the dataset under investigation (due to the size of the dataset and the observation of normality in all but one condition). This has been clarified in both the methods section 2.4 and the results section 3.3.

The bar charts are widely used in most scientific papers, but this is inappropriate in terms of statistics. Instead, a dot plot, boxes with whiskers, or the points showing the mean values with the error bars for standard deviation should be used. Please change the graph type in all the figures due to my suggestions.

Due to our relatively low number of experimental repeats, box plots are not informative in this case. Instead, we have retained bar charts but have also shown the individual data points alongside the bars in the results section 3.2 and 3,3 (figures 2 and 3). This provides all experimental data and so we hope that this suitably addresses the point raised by the reviewer.

The Authors have to specify if the error bars on the figures present the standard deviations (SD) or standard errors of the mean (SEM). From a substantive point of view the error bars should present the SD. If the error bars show the SEM, then it should be changed to SD.

We have now specified that the errors bars shown on the figures represent the standard deviation (see the figure legends of all figures). We thank the reviewer for highlighting this oversight.

Two biological repeats is not enough to perform a reliable statistical test. Three biological repeats is the absolute minimum. The Authors have to perform one more biological repeat wherever there are two biological repeats.

We agree with the reviewer that two biological repeats do not provide robust statistical data and our results should therefore be viewed as preliminary/exploratory. As discussed above, we are sadly unable to perform further experiments at present. Instead, we have addressed this point in a number of ways.

1. We have reframed the discussion within our manuscript in numerous locations to more clearly present our work as exploratory and to frame our conclusions as preliminary / proof-of-concept. We believe our data has value and is appropriate for publication when pitched at this level.

2. We have shown individual data points on Figures 1 and 2 to better visualise variability within our experiments.

3. We have performed further analyses to assess correlations between our fluorescence and TEER measurements. As for the standalone fluorescence plots, these analyses show statistically significant results, demonstrating correlations between fluorescence intensities and TEER values (see section 3.4, Figure 4 and Supplementary Figures). While these results should still be viewed as exploratory due to the low number of experimental repeats, this nonetheless further demonstrates that we have observed clear effects in this study (i.e. with effect sizes high enough to generate statistical significance even with a low number of measurements).

Together, we believe this addresses the reviewer’s point and justifies publication of our preliminary data at this stage. Importantly, this will help to support and enable further investigation of the use of multiple fluorescent agents for epithelial barrier assessment, which in turn will further validate our proof-of-concept results.

Editorial issues (page numbering applies to the entire Manuscript Draft document):

Correct the reference number 19 and 23. Number 19: “ACS Paper” – this information is not enough. Number 23: the reference is split into two paragraphs, thus merge these two paragraphs into one. Page 11, line 5: change “However, While” to “However, while” (probably typo). English language comments Page 10, third line in “Introduction”: to avoid unnecessary repetitions, change “and active and passive immunity” to “as well as active and passive immunity”.

We thank the reviewer for highlighting these points. We have corrected the editorial issues as requested.

Attachment Submitted filename: Itemised Response to Reviewers PLOS One.docx

# Peer review: Decision Letter 1

**DOI:** 10.1371/journal.pone.0358746.r003

**Authors:** Mária Deli

7 Sep 2026

Assessment of epithelial barrier integrity in cellular monolayer models using low cost, clinically approved fluorescent contrast agents

PONE-D-25-52156R1

Dear Dr. Watson,

We’re pleased to inform you that your manuscript has been judged scientifically suitable for publication and will be formally accepted for publication once it meets all outstanding technical requirements.

Within one week, you’ll receive an e-mail detailing the required amendments. When these have been addressed, you’ll receive a formal acceptance letter and your manuscript will be scheduled for publication.

An invoice will be generated when your article is formally accepted. Please note, if your institution has a publishing partnership with PLOS and your article meets the relevant criteria, all or part of your publication costs will be covered. Please make sure your user information is up-to-date by logging into Editorial Manager at Editorial Manager® and clicking the ‘Update My Information' link at the top of the page. For questions related to billing, please contact billing support.

If your institution or institutions have a press office, please notify them about your upcoming paper to help maximize its impact. If they’ll be preparing press materials, please inform our press team as soon as possible -- no later than 48 hours after receiving the formal acceptance. Your manuscript will remain under strict press embargo until 2 pm Eastern Time on the date of publication. For more information, please contact onepress@plos.org.

Kind regards,

Mária A. Deli, M.D., Ph.D.

Academic Editor

PLOS One

Additional Editor Comments (optional):

Reviewers' comments:

Reviewer's Responses to Questions

**Comments to the Author**

Reviewer #1: All comments have been addressed

Reviewer #2: All comments have been addressed

**********

2. Is the manuscript technically sound, and do the data support the conclusions?

Reviewer #1: Yes

Reviewer #2: Yes

**********

3. Has the statistical analysis been performed appropriately and rigorously?

Reviewer #1: Yes

Reviewer #2: Yes

**********

4. Have the authors made all data underlying the findings in their manuscript fully available?

The PLOS Data policy

Reviewer #1: Yes

Reviewer #2: Yes

**********

5. Is the manuscript presented in an intelligible fashion and written in standard English?

Reviewer #1: Yes

Reviewer #2: Yes

**********

Reviewer #1: (No Response)

Reviewer #2: The Authors have accurately addressed my suggestions. The manuscript no. PONE-D-25-52156R1 is ready for publication in PLOS One.

**********

what does this mean?). If published, this will include your full peer review and any attached files.

If you choose “no”, your identity will remain anonymous but your review may still be made public.

**Do you want your identity to be public for this peer review?**  For information about this choice, including consent withdrawal, please see our Privacy Policy

Reviewer #1: No

Reviewer #2: **Yes:**  Radoslaw Bednarek

**********

# Editor report: Acceptance letter

**DOI:** 10.1371/journal.pone.0358746.r004

**Authors:** Mária Deli

PONE-D-25-52156R1

PLOS One

Dear Dr. Watson,

I'm pleased to inform you that your manuscript has been deemed suitable for publication in PLOS One. Congratulations! Your manuscript is now being handed over to our production team.

At this stage, our production department will prepare your paper for publication. This includes ensuring the following:

* All references, tables, and figures are properly cited

* All relevant supporting information is included in the manuscript submission,

* There are no issues that prevent the paper from being properly typeset

You will receive further instructions from the production team, including instructions on how to review your proof when it is ready. Please keep in mind that we are working through a large volume of accepted articles, so please give us a few days to review your paper and let you know the next and final steps.

Lastly, if your institution or institutions have a press office, please let them know about your upcoming paper now to help maximize its impact. If they'll be preparing press materials, please inform our press team within the next 48 hours. Your manuscript will remain under strict press embargo until 2 pm Eastern Time on the date of publication. For more information, please contact onepress@plos.org.

You will receive an invoice from PLOS for your publication fee after your manuscript has reached the completed accept phase. If you receive an email requesting payment before acceptance or for any other service, this may be a phishing scheme. Learn how to identify phishing emails and protect your accounts at https://explore.plos.org/phishing.

If we can help with anything else, please email us at customercare@plos.org.

Thank you for submitting your work to PLOS One and supporting open access.

Kind regards,

PLOS One Editorial Office Staff

on behalf of

Prof. Mária A. Deli

Academic Editor

PLOS One
